# Apport de l’épreuve d’effort dans la prise en charge des cardiopathies ischémiques

**DOI:** 10.11604/pamj.2018.31.229.15927

**Published:** 2018-12-12

**Authors:** Joel Bamouni, Dangwé Temoua Naibe, Relwendé Aristide Yameogo, Dakaboué Germain Mandi, Georges Rosario Christian Millogo, Nobila Valentin Yameogo, Jonas Koudougou Kologo, Anna Thiam-Tall, Lucie Adélaïde Valérie Nébié, Patrice Zabsonré

**Affiliations:** 1 Service de Médecine, CHU de Ouahigouya, Burkina Faso; 2 Faculté de Médecine, Centre Universitaire Polytechnique de Ouahigouya, Burkina Faso; 3 Hôpital Général de Référence Nationale de N’Djamena, N’Djamena, Tchad; 4 Université de N’Djamena, N’Djamena, Tchad; 5 Normandie Université, UNIHAVRE- UNIROUEN - UNICAEN, CNRS, UMR IDEES Le Havre, France; 6 Service de Cardiologie - CHU Yalgado Ouédraogo, Ouagadougou, Burkina Faso; 7 Unité de Formation et de Recherche en Sciences de la Santé, Université Ouaga I: Professeur Joseph Ki-Zerbo, Ouagadougou, Burkina Faso; 8 Polyclinique Internationale de Ouagadougou, Burkina Faso

**Keywords:** Epreuve d´effort, protocole de Bruce, cardiopathies ischémiques, Burkina Faso, Stress test, Bruce protocol, ischemic heart disease, Burkina Faso

## Abstract

L’épreuve d’effort est un moyen diagnostic utile pour les cas de suspicion d’angor avec une faible sensibilité mais une bonne spécificité. Elle est également très utile dans l’évaluation du risque, de l’efficacité du traitement et la guidance des prescriptions médicales après contrôle des symptômes d’ischémie. L’objectif de notre travail était d’analyser l’apport de l’épreuve d’effort à la prise en charge des cardiopathies ischémiques dans le Service de Cardiologie du CHU YO. Il s’est agi d’une étude rétrospective sur 60 patients ayant bénéficié d’une épreuve d’effort de janvier 2012 à décembre 2013. L’épreuve d’effort a été réalisée sur tapis roulant selon le protocole de Bruce modifié. Soixante patients ont bénéficié d’une épreuve d’effort durant notre période d’étude. L’âge moyen des patients était de 49 ± 10,8 ans. Le sex-ratio était de 1,2. Tous les patients avaient effectué l’épreuve d’effort sur tapis roulant. Un antécédent de coronaropathie était noté chez 22 patients. La recherche d’une insuffisance coronarienne était l’indication de l’épreuve d’effort dans 83% des cas. Elle était démaquillée dans 78% des cas. L’épreuve d’effort était arrêtée pour effort maximal dans 46 cas (soit 77%). La durée moyenne de l’effort était de 11,7 mn ± 3,2. Dix pour cent des patients avaient eu une épreuve d’effort positive et 10% une épreuve d’effort litigieuse. Notre étude va en outre contribuer à vulgariser davantage cet examen qui reste peu prescrit dans notre environnement et même parmi les médecins cardiologues. Cependant, des efforts restent à faire afin d’améliorer la qualité de la pratique de cet important examen dans la prise en charge des coronaropathies dans un contexte de pays à ressources limitées.

## Abstract (en)

Stress test is a useful diagnostic tool in patients with suspected angina pectoris with low sensitivity but high specificity. It is also very useful in the evaluation of the risk, of the effectiveness of treatment and it is a useful guidance on medical prescriptions after controlling the symptoms of ischemia. This study aims to analyze the contribution of stress test to the treatment of ischemic heart disease in the Department of Cardiology at the University Hospital CHU YO. We conducted a retrospective study of 60 patients who had undergone stress test from January 2012 to December 2013. Stress test was performed using a treadmill according to the modified Bruce protocol. Sixty patients underwent stress test during the study period. The average age of patients was 49± 10.8 years. Sex-ratio was 1.2. All patients underwent treadmill stress test. Twenty-two patients had a history of coronary artery disease. Estimating presence of coronary artery disease was the indication for stress test in 83% of cases. It was detected in 78% of cases. Stress test was stopped when maximal workload was reached in 46 cases (or 77%). Mean exercise duration was 11,7 mn ± 3,2. Ten percent of patients had a positive stress test and 10% a dubious test. Our study will also contribute to transfer knowledge on this diagnostic test which is still little prescribed in our environment and even among cardiologists. However, efforts should be made in order to improve the quality of stress test practice in the management of coronary artery disease in a context of countries with limited resources.

## Introduction

L’épreuve d’effort (EE) est l'enregistrement de l'activité électrique du cœur au cours d'un effort physique (sur tapis roulant ou sur bicyclette ergométrique) [1]. Elle reste une exploration cardio-vasculaire non invasive de choix (souvent couplée à la scintigraphie myocardique) dans le diagnostic et le suivi des insuffisances coronariennes [2] dans notre contexte de pays en développement où la coronarographie reste encore peu accessible. L’épreuve d’effort reste une exploration non invasive du cœur peu connue, même au sein des praticiens. On observe une régression de sa pratique au profit de la coronarographie et dans certains cas de l’échographie de stress dans les pays développés. Cependant, dans nos pays à ressources limitées et où les revenus sont généralement modestes, quelle place devrait occuper l’EE eu égard au fait qu’elle est non invasive, moins coûteuse, de réalisation plus facile et surtout en l’absence de bloc de coronarographie. C’est un moyen diagnostic utile pour les cas de suspicion d’angor avec une faible sensibilité mais une bonne spécificité [3]. L’EE est également très utile dans l’évaluation du risque, de l’efficacité du traitement et la guidance des prescriptions médicales après contrôle des symptômes d’ischémie [3]. Au Burkina Faso, c’est en janvier 2012 que les épreuves d’effort ont été réalisées dans un hôpital public dans le Service de Médecine Nucléaire du Centre Hospitalier Universitaire Yalgado Ouédraogo (CHU YO) en collaboration avec le service de Cardiologie. Depuis lors, l’activité est menée régulièrement. Cependant, un bilan de l’apport de celle-ci dans la prise en charge des pathologies cardiovasculaires n’a pas encore été fait. Notre étude se propose d’analyser l’apport de l’épreuve d’effort à la prise en charge des cardiopathies ischémiques dans le Service de Cardiologie du CHU YO.

## Méthodes

### Cadre de l’étude

Notre étude a eu pour cadre le service de Médecine Nucléaire du Centre Hospitalier Universitaire Yalgado Ouédraogo (CHU YO). Il constitue pour le moment le seul service de médecine nucléaire du Burkina Faso. Le Service de Médecine Nucléaire dispose d’un tapis roulant pour l’épreuve d’effort. Tous les examens sont réalisés par des cardiologues.

### Période et type d’étude

Il s’est agi d’une étude rétrospective, descriptive effectuée sur l’analyse des comptes rendus d’épreuve d’efforts réalisés de janvier 2012 à décembre 2013 dans le Service de Médecine Nucléaire.

### Échantillonnage

Nous avons inclus tous les comptes rendus des patients adressés pour épreuve d’effort quel que soit l’indication. Nous avons exclu de notre étude tous les comptes rendus inexploitables du fait de manque d’informations essentielles telles que l’âge, le sexe, l’indication de l’examen, les résultats de l’épreuve d’effort ou la conclusion à la fin du compte rendu.

### Réalisation de l’examen

L’épreuve d’effort a été réalisée sur tapis roulant. Le protocole utilisé était celui de Bruce modifié. Il s’agissait donc d’une épreuve triangulaire où la charge était augmentée de manière progressive toutes les trois minutes. La prise de la tension artérielle était faite manuellement chaque trois minutes. L’épreuve d’effort était arrêtée si le patient présentait des signes de positivité, des signes fonctionnels invalidants ou un épuisement. Les critères de positivité de l’EE étaient: le sous-décalage de ST de plus de 1mm, situé de 0.06 à 0.08 seconde après le point J, horizontal ou descendant. Le territoire du sous- décalage n’a pas de valeur topographique; le sus-décalage de ST en l’absence d’infarctus. Il témoigne en général d’une ischémie transmurale; des modifications de l’onde R, inversion de l’onde U ou triplement de l’onde T [4]. L’épreuve d’effort était dite litigieuse s’il s’agissait: d’une épreuve d’effort sous maximale c'est-à-dire la fréquence maximale théorique (FMT) et/ou le niveau d’effort cible ne sont pas atteints; d’un sous-décalage ascendant; d’un sous-décalage présent seulement en phase de récupération; d’une épreuve positive cliniquement sans anomalie électrique; de la présence d’anomalies de la repolarisation à l’état initial, de cicatrice d’infarctus, de bloc de branche gauche, de bloc de branche droit, de Wolff Parkinson White, d’un pacemaker, de signes d’hypertrophie ventriculaire gauche [4].

### Définitions opérationnelles

La fréquence maximale théorique (FMT) a été appréciée à partir de la formule d’Astrand (la plus utilisée) FMT = 220 - âge du sujet ± 10. Pour les patients cardiaques, notamment ceux traités par bêtabloquants ou autres traitements ralentisseurs, nous avons utilisé la formule de Brawner FMT = 164 – 0,7×âge [2, 5]. Le niveau d’effort atteint a été défini par le pourcentage de FMT atteint [2, 5]: effort sous- maximal si FC maximale < 85% FMT, effort sub- maximal si 85% FMT ≤ FC maximale < 100% FMT, effort maximal si FC maximal ≥ 100% FMT.

L’épreuve d’effort était dite « démaquillée » lorsque nous arrêtons les médicaments anti-angineux, donnés souvent en anticipation pour confirmer ou infirmer le diagnostic d’insuffisance coronaire et pour évaluer les performances du patient [4].

### Collecte des données

Les informations ont été recueillies à l’aide d’une fiche d’enquête qui a été remplie par les cardiologues. Cette fiche comportait des paramètres à étudier. Nous avons collecté les données socio-démographiques (âge, le sexe, la profession, la résidence), les données techniques (ergomètre, le protocole, le prescripteur) et les données cliniques et paracliniques (les antécédents du patients, les facteurs de risque cardiovasculaire, le traitement en cours, l’indication, les résultats de l’épreuve d’effort au repos et à l’effort).

### Analyse des données

Les données ont été analysées à l’aide du logiciel Epi Info 7. L’analyse statistique a été effectuée avec le test exact de Fischer pour un seuil de significativité à 5% pour la comparaison des proportions. La comparaison des moyennes a été effectuée à l’aide d’ANOVA. Les données ont été anonymisées avant toutes manipulations. Nous avons reçu l’autorisation de la commission médicale d’établissement pour la réalisation de l’étude.

## Résultats

Durant la période d’étude, 60 épreuves d’effort ont été réalisées dans le service.

### Caractéristiques socio-démographiques

**Figure 1:**
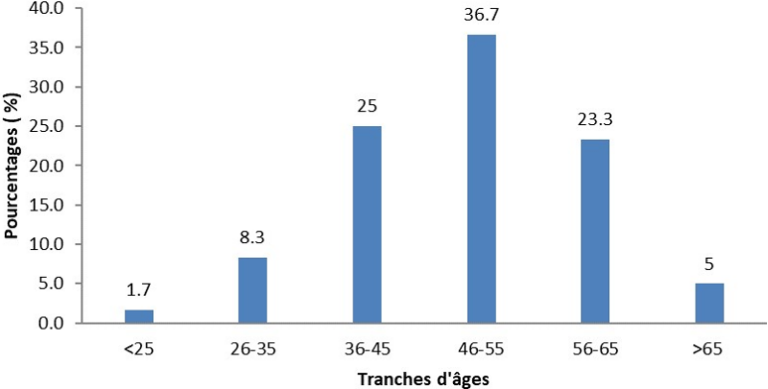
Répartition des patients selon les tranches d’âge

L’âge moyen était de 49 ± 10,8 ans avec des extrêmes de 13 et 73 ans. La classe modale était celle de 46 - 55 ans avec une proportion de 37%. La Figure 1 montre la répartition des patients selon les tranches d’âge. La moyenne d’âge des hommes était de 50,7 ± 12,2 ans avec des extrêmes de 13 et 73 ans. La moyenne d’âge des femmes était de 47,2 ± 8,5 ans avec des extrêmes de 31 et 63 ans. La différence n’était pas statistiquement significative (p = 0,21). Le sex ratio était de 1,2 soit 33 hommes et 27 femmes. Tous les patients résidaient dans la ville de Ouagadougou et 67% étaient des fonctionnaires. Le Tableau 1 montre la répartition des patients en fonction de la profession.

**Tableau 1:** Répartition des patients en fonction de la profession

	Effectif (n)	Pourcentage (%)
Fonctionnaire	40	67
Retraité	06	10
Élève/ Étudiant	02	03
Commerçant	03	05
Femme au foyer / Cultivateur	06	10
Militaire	02	03
Religieux	01	02
Total	60	100

### Les données cliniques

#### Antécédents médicaux

On notait un antécédent de coronaropathie dans 13 cas (22%). Il s’agissait d’un angor stable dans un cas et d’une cardiopathie ischémique dans 12 cas. Une hypertension artérielle était notée dans 44 cas (38%) et une obésité dans 23 cas (20%). Neuf patients ne présentaient aucun facteur de risque cardiovasculaire. Le Tableau 2 montre la répartition des patients en fonction des facteurs de risque cardiovasculaire.

**Tableau 2:** Répartition des patients en fonction des facteurs de risque cardiovasculaire

	Effectif (n)	Pourcentage (%)
HTA	44	38
Diabète	10	8
Tabac	10	8,5
Sédentarité	11	09
Dyslipidémie	19	16
Obésité	23	20

#### Traitement en cours

Dans les traitements en cours des patients, les familles de médicaments les plus utilisées étaient les antiagrégants plaquettaires (62% des cas), les inhibiteurs de l’enzyme de conversion/ antagonistes des récepteurs de l’angiotensine 2 (IEC/ARA2) (45% des cas) et les bêtabloqueurs (43% des cas). Le Tableau 3 montre la répartition des patients selon les traitements en cours.

**Tableau 3:** Répartition des patients en fonction du traitement en cours

	Effectif (n)	Pourcentage (%)
Amiodarone	01	02
Calcibloqueurs	13	22
Bêtabloqueurs	26	43
IEC/ARA2	27	45
Dérivés nitrés	15	25
Statines	23	38
Antiagrégants plaquettaires	37	62
Anticoagulants	04	07

#### Prescripteurs et indications

L’épreuve d’effort a été prescrite dans tous les cas par des médecins cardiologues de la ville de Ouagadougou. Dans notre étude, l’épreuve d’effort était prescrite pour la recherche d’une insuffisance coronarienne dans 50 cas (83%), pour le suivi d’un infarctus du myocarde dans huit cas (soit 13%) et pour troubles du rythme cardiaque dans deux cas (soit 3%). Ces indications correspondaient sur le plan fonctionnel aux symptômes tels que libellés dans le Tableau 4.

**Tableau 4:** Répartition des patients en fonction des symptômes

	Effectif (n)	Pourcentage (%)
Douleur thoracique	50	83
Dyspnée	33	55
Douleur thoracique + dyspnée	13	22
Palpitations	02	03

#### Épreuve démaquillée

L’épreuve d’effort était démaquillée dans 47 cas (78%) avant l’épreuve d’effort. Cet arrêt thérapeutique a duré 48 heures dans 34 cas (72%) et 24 heures dans 13 cas (28%). Les arrêts de 24 heures ont concerné les statines, les antiagrégants plaquettaires.

### Données paracliniques

#### ECG de repos

La moyenne des fréquences cardiaques (FC) de repos des 60 patients était de 89 bpm ± 13,9 bpm avec des extrêmes de 52 et 139 bpm. L’ischémie était observée dans 39 cas (65%). Le Tableau 5 montre la répartition des patients selon les signes électriques à l’ECG de repos.

**Tableau 5:** Répartition des patients en fonction des signes électriques à l'ECG

	Effectif (n)	Pourcentage (%)
Ischémie	39	65
Lésion	02	3,3
Nécrose	13	21,6
Normal	12	20

#### Épreuve d’effort

**Figure 2:**
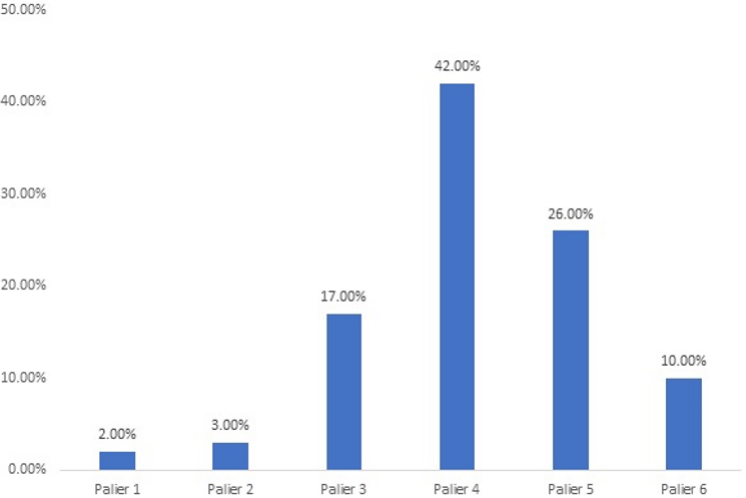
Répartition des patients en fonction du palier atteint

La durée moyenne de l’épreuve d’effort (EE) était de 11,7 mn ± 3,2 avec des extrêmes d’une mn et 17 mn. La moyenne des consommations maximales d’oxygène des 60 patients était de 30,4 ml/kg/mn ± 12,5 avec des extrêmes de 8 et 61 ml/kg/mn. La moyenne des *metabolic equivalent of the task* (METs) des patients était de 8,8 ml/mn/kg d’O2 ± 3,2 avec des extrêmes de 3 et 17 ml/mn/kg (1 MET = 3,5 ml d’O2/Kg/mn au repos: métabolisme basal) Quarante-deux patients soit 70% ont atteint un effort sub-maximal, onze soit 18%, un effort maximal et sept soit 12%, un effort sous maximal. Le nombre moyen de paliers atteints était de 4 ± 1 avec des extrêmes d’un et six. Dans 25 cas (42%), le palier 4 avait été atteint. La Figure 2 montre la répartition des patients en fonction du nombre de paliers atteints. La moyenne de la pression artérielle systolique (PAS) au repos des patients était de 129mmHg ± 13 avec des extrêmes de 100mmHg et 160mmHg. Celle de la pression artérielle diastolique (PAD) au repos des patients était de 83mm g ± 11,4 avec des extrêmes de 60mmHg et 110mmHg.

**Figure 3:**
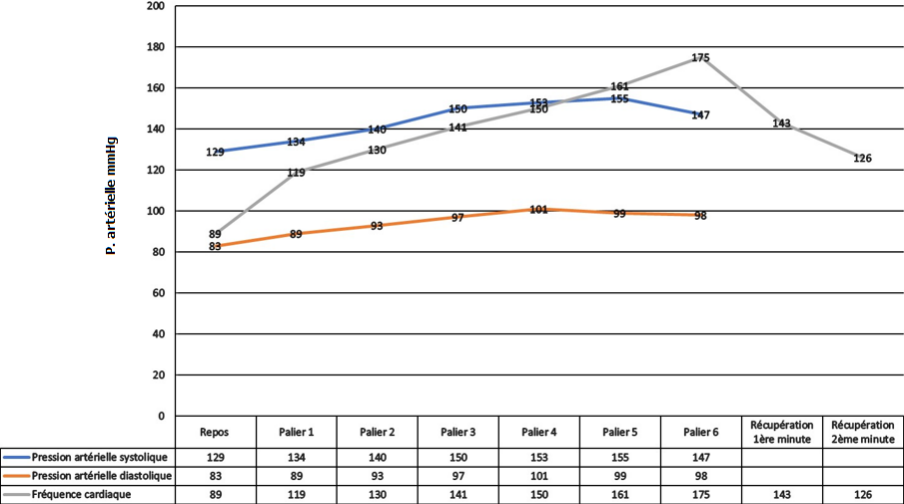
évolution de la pression artérielle diastolique, systolique et de la fréquence cardiaque au cours de l’épreuve d’effort

La moyenne des PAS maximales à l’effort des patients était de 156mmHg ± 21 avec des extrêmes de 110mmHg et 240mmHg. Celle des PAD maximales à l’effort était de 101mmHg ± 13 avec des extrêmes de 70mmHg et 120mmHg. La FC moyenne au repos était de 89 battements par minute (bpm) ± 14 bpm avec des extrêmes de 52 et 139 bpm. Au maximum de l’effort elle était de 157 ± 18 avec des extrêmes de 113 et 190 bpm. La fréquence cardiaque augmentait de manière linéaire avec l’effort. La moyenne des FC à une minute de récupération était de 143 bpm ± 18 avec des extrêmes de 99 bpm et 176 bpm et à deux minutes de 129 bpm ± 18 avec des extrêmes de 85 bpm et 170 bpm. La PAS augmentait de manière plus ou moins linéaire avec l’effort. Une baisse de la PAS a été observée à partir du palier 5. La PAD augmentait au début de l’effort puis plafonnait à 95 mm Hg. La Figure 3 montre l’évolution des PAS, PAD et FC au cours de l’EE. Le critère d’arrêt était l’effort maximal dans 46 cas (77%). Le Tableau 6 montre les différents critères d’arrêt de l’EE. L’EE était positive dans six cas (10%), négative dans 46 cas (80%) et litigieuse dans six cas (10%). Au cours de l’EE, 20 patients (33%) ont présenté des signes fonctionnels. Le Tableau 7 montre la répartition des patients ayant présenté des signes fonctionnels au cours de l’EE.

**Tableau 6:** Répartition des patients en fonction des critères d'arrêt de l'EE

	Effectif (n)	Pourcentage (%)
Effort maximal	46	77
Épuisement	07	11
EE positive	06	10
TDR grave	01	02
Total	60	100

**Tableau 7:** Répartition des patients ayant présenté des signes fonctionnels au cours de l'EE

	Effectif (n)	Pourcentage (%)
Douleurs thoraciques	05	25
Dyspnée	03	15
Fatigue	08	40
Palpitations	04	20
Total	20	100

## Discussion

Notre étude retrouvait une moyenne d’âge de 49 ± 10,8 ans. Les études réalisées sur l’épreuve d’effort [6–8] retrouvaient des moyennes d’âge similaire à la nôtre. L’indication de l’épreuve d’effort dans ces différents travaux était essentiellement pour un bilan coronarien comme dans notre étude (83%). Pour toutes ces séries comme la nôtre, l’âge moyen était supérieur à 40 ans. En effet la prévalence de la pathologie cardiovasculaire et en particulier coronaire est faible chez le sujet jeune. Dans notre étude, 12 patients (soit 20%) avaient des antécédents d’infarctus du myocarde. Ce taux est nettement plus élevé que ceux rapportés par Tagny [9] (6,7%) et Høilund-Carlsen *et al.* [10] (8%). Les recommandations sont unanimes sur le fait que le post infarctus du myocarde est une indication majeure de l’EE cardiologique. Elle permet de rechercher une ischémie résiduelle, des troubles du rythme et d’évaluer le traitement [3, 11].

Dans notre étude, l’EE était prescrite pour recherche d’une insuffisance coronarienne dans 58 cas (soit 97%). Dans la série de Tahirou *et al.* [6] le bilan coronarien était également l’indication majeure dans 84% des cas. D’une manière générale ces résultats concordent avec les données de la littérature. Dans la majorité des études en effet, l’indication majeure du test d’effort reste le bilan coronarien [6, 7, 9, 12]. Le tapis roulant a été utilisé dans notre étude. Dans la littérature, les équipes utilisaient soit le tapis roulant [13–15] pour la réalisation de l’EE. Le tapis roulant semble être l’ergomètre de premier choix. Le test est plus physiologique et le patient se fatigue moins rapidement. Ce qui permet d’atteindre les objectifs de fréquence cible plus souvent [1]. Le bicycle ergométrique semble plus indiqué en cas de contre-indications au tapis roulant et à certaines indications bien précises notamment pour l’évaluation de la pression artérielle et pour des EE rythmologiques. Chez nos patients, la prise de la pression artérielle était manuelle. Dans la majorité des études notamment celle de Wielemborek *et al.* [16] en Pologne la prise de la pression artérielle était également manuelle. Furtado *et al.* [17] ont comparé dans une étude la prise de la PA manuelle et automatique à l’effort. Ils ont conclu que les mesures prises par les deux méthodes étaient similaires et reproductibles. En général dans la littérature, la prise manuelle est recommandée car elle est moins source d’erreur surtout chez un patient en mouvement.

Dans notre étude, 88% des patients avaient une FMT ≥ 85%. Ce taux était de 43% dans la série de Tahirou *et al.* [6] au Niger et 63% dans celle de Tagny [9] en Côte d’Ivoire. Ces taux d’effort maximal nettement plus bas que celui de notre série s’expliquerait en partie par le type d’ergomètre utilisé (tapis roulant dans notre étude, bicycle ergométrique pour les séries du Niger et de la Côte d’Ivoire) et le protocole choisi. Sous ergocycle le test est moins physiologique et les patients se fatiguent plus rapidement ne permettant donc pas l’atteinte de la fréquence cible [1]. En effet, dans notre étude, le critère d’arrêt était l’effort maximal dans 77% et l’épuisement musculaire dans 62% des cas dans les séries de Tahirou [6] et Tagny [9]. La durée moyenne de l’EE était de 11,7 mn ± 3,2 avec des extrêmes d’une mn et 17 mn dans notre étude. Ces résultats sont comparables aux données de la littérature. Toutes les recommandations s’accordent à dire que la durée idéale de l’effort doit se situer entre 8 et 15 minutes. En dessous de 8 minutes la sollicitation myocardique est rarement suffisante, le facteur limitant étant la musculature périphérique. S’il dure plus de 15 minutes, c’est surtout la capacité d’endurance qui est testée. Dans les deux cas, la sensibilité du test pour la détection de l’ischémie est diminuée [18]. Notre étude a eu les limites inhérentes à toute étude rétrospective. La nature rétrospective de notre étude ne nous a pas permis d’avoir des renseignements concernant le caractère précis des douleurs thoraciques qui étaient l’indication majeure. Par conséquent la probabilité pré-test de chaque patient n’a pas été estimée. Aucun de nos patients ayant eu un test positif n’a bénéficié d’une coronarographie. L’identification des faux positifs et des vrais positifs, et l’évaluation de la sensibilité et de la spécificité du test ne figurent donc pas dans nos données.

## Conclusion

Les insuffisances coronariennes occuperont une place de plus en plus importante dans la pathologie cardio-vasculaire dans les pays en développement. Ceci eu égard à l'augmentation de l'espérance de vie et au cumul des facteurs de risque cardio-vasculaire. Dans le même temps, les moyens diagnostiques restent insuffisants. Notre étude montre l'importance de l'épreuve d'effort dans le diagnostic et partant dans le traitement de ces pathologies en attendant de meilleurs moyens tels que la coronarographie. Son intérêt est à la fois diagnostic, pronostic et ses indications variées. Il existe des contre-indications à connaître afin d’éviter des complications potentiellement dangereuses pour le patient. Notre étude va en outre contribuer à vulgariser davantage cet examen qui reste peu prescrit dans notre environnement et même parmi les médecins cardiologues. Cependant, des efforts restent à faire afin d’améliorer la qualité de la pratique de cet important examen dans la prise en charge des coronaropathies dans un contexte de pays à ressources limitées.

### Etat des connaissances actuelles sur le sujet

- L’épreuve d’effort est utile pour le diagnostic et le suivi des cardiopathies ischémiques;
- Couplée à la scintigraphie, elle donne des meilleurs résultats pour l’évaluation des cardiopathies ischémiques.

### Contribution de notre étude à la connaissance

- L’examen est très peu prescrit par les cardiologues;
- L’épreuve d’effort constitue un outil très important dans la prise en charge des patients coronariens dans notre contexte.

## Conflits des intérêts

Les auteurs ne déclarent aucun conflit d'intérêts.

## Remerciements

Aux personnels du Service de Médecine Nucléaire pour leur collaboration.

## References

[1] Cohen-Solal A, Carré F (2009). Guide pratique des épreuves d’effort cardiorespiratoires.

[2] Zerbib E (2012). Explorations radio-isotopiques dans la maladie coronarienne. EMC - Cardiol-Angéiologie.

[3] Task Force Members, Montalescot G, Sechtem U (2013). 2013 ESC guidelines on the management of stable coronary artery disease: the Task Force on the management of stable coronary artery disease of the European Society of Cardiology. Eur Heart J.

[4] Marcadet D-M (2004). Électrocardiogramme d’effort. EMC - Cardiol-Angéiologie.

[5] Wiel E, Assez N, Goldstein P (2012). Stratégie de prise en charge des syndromes coronariens aigus. EMC - Médecine Urgence.

[6] Tahirou I, Moussa IDJ, Ada A (2012). Analyse des résultats préliminaires de scintigraphie myocardique réalisée à l’institut des radio-isotopes (IRI) du Niger. À propos de 37 cas. Médecine Nucl.

[7] Sfar R, Khlifi H, Kamoun T (2013). Douleurs thoraciques et scintigraphie myocardique: à propos de 171 cas. Médecine Nucl.

[8] Ait idir M, Guensi A, Taleb S (2013). Ischémie myocardique du diabétique: apports de la scintigraphie myocardique de perfusion. Médecine Nucl.

[9] Tagny SJH (2011). Bilan d’activité de l’épreuve d’effort à l’institut de cardiologie d’Abidjan de Janvier 2008 à Avril 2010.

[10] Høilund-Carlsen PF, Johansen A, Christensen HW (2005). Usefulness of the exercise electrocardiogram in diagnosing ischemic or coronary heart disease in patients with chest pain. Am J Cardiol.

[11] Fihn SD, Gardin JM, Abrams J (2012). ACCF/AHA/ACP/AATS/PCNA/SCAI/STS Guideline for the Diagnosis and Management of Patients With Stable Ischemic Heart Disease: A Report of the American College of Cardiology Foundation/American Heart Association Task Force on Practice Guidelines, and the American College of Physicians, American Association for Thoracic Surgery, Preventive Cardiovascular Nurses Association, Society for Cardiovascular Angiography and Interventions, and Society of Thoracic Surgeons. J Am Coll Cardiol.

[12] Kósa I, Vassányi I, Nemes A (2011). Stress ECG utilization in the evaluation of patients with chest paIn: The real practice in Hungary with 10million inhabitants. Int J Cardiol.

[13] Fadayomi MO, Akinroye KK (1987). Implications of positive treadmill exercise tests in asymptomatic adult African blacks. Eur Heart J.

[14] Chalela WA, Fukushima RB, Araujo F (2009). Treadmill exercise testing of asymptomatic men and women without evidence of heart disease. Braz J Med Biol Res Rev Bras Pesqui Medicas E Biol.

[15] Michaelides AP, Papapetrou D, Aigyptiadou M-NK (2004). Detection of multivessel disease post myocardial infarction using an exercise-induced QRS score. Ann Noninvasive Electrocardiol Off J Int Soc Holter Noninvasive Electrocardiol Inc.

[16] Wielemborek-Musia K, Kaleta D, Jegier A (2005). [The blood pressure response to physical exertion in adults: a preliminary survey results]. Przegl Lek.

[17] Furtado EC, Ramos P dos S, de Araújo CGS (2009). Blood pressure measurement during aerobic exercise: subsidies for cardiac rehabilitation. Arq Bras Cardiol.

[18] Weber R (1999). Détection de l’ischémie myocardique par l’ergométrie conventionnelle. Médecine Hygiène.

